# Cholera

**Published:** 1873-08

**Authors:** 


### CHOLERA.

Cholera, as at present accepted, is of the nature of organic matter, always in existence somewhere, and its history, contemporary with the history of India, of which country it is a native. The present doctrine of its pathology is, that *it essentially consists of a poison that has been absorbed or introduced into the blood of the living body; that this poisonous cholera seed there undergoes speedy and enormous multiplication, and produces all of the symptoms and changes which, in the aggregate, constitute a case of cholera.* If it be true, and we believe it is, that with the progress of civilization the intellectual qualities of mankind at large are elevated as the health of the body is protected, and the physical frame invigorated, then surely much of the human advancement during the last fifty years is due to our better acquaintance with the nature of cholera. However lamentable the devastations of cholera since its exodus from India, in 1831, the devotees of sanitary science have used well their opportunities; and, if the people at large have not profited by the truths which have been worked out, and which it is our present purpose to concisely reiterate, *the fault lies at their own doors.*

The general conclusions of experimental and other researches in regard to the origin and propagation of cholera, may be summarized as follows:

1. Cholera may be produced by the direct introduction of the poison into the blood.

2. *All of the evacutions of persons affected with cholera are dangerous,* and capable of communicating the disease; that this dangerous property of the evacuations is not only possessed by the discharges from the bowels, but, at least equally, by *the vomit and the urine.*

3. The infecting power of the discharges is in proportion to their freshness.

4. When the discharges have undergone decomposition—to which they are exceedingly prone—or are allowed to dry on the clothing, to remain in the apartment, or are thrown out without disinfection, the disease is spread with more facility; but the symptoms are less distinct, being complicated with those of poisoning by the accompanying putrid materials.

5. After the introduction of cholera poison into the animal system, *two or three days* usually elapse before the characteristic symptoms are produced.

6. Cholera is distributed and diffused by *the neglected evacuations of persons affected with it;* by these discharges becoming mixed with the drinking water in rivers and wells; by cholera soiled clothing, ships, and other vehicles of travel related to human intercourse, and by "*localizing conditions*"—which usually account for the apparently erratic course of cholera.

No truth is better established than that *dirt* and *impurity* are potent instrumentalities for the propagation of cholera. *Dirt*, *danger*, *disease*, and *death*, form an alliterative series of consequences of momentous importance at the present time, to almost every populous community in the United States. For the denizens of the foul cellars and other tenements, in New York, which are at the time of this writing being thrust out, and made "conspicuous examples" of sanitary work, there is much room for commiseration. *They* are not the true criminals. It is related of a London magistrate, about thirty years ago, when the standard of public health in London was about the same as it is at the present time in New York, on having to exercise his functions in regard to a low tenement, "I have often said," he observed, "that if empty casks were placed along the streets of Whitechapel, in a few days each of them would have a tenant; and these tenants would keep up their kind, and prey upon the rest of the community. I am sure that if *such facilities were offered*, there is no conceivable degradation to which portions of the species might not be reduced."

The few villainous cellars that have been untenanted lately by the board of health in New York, constitute an exceedingly small proportion of the other facilities offered for the same class of tenants—not only in New York and Brooklyn, but in various others of our large cities—tolerating and holding out inducements to cholera all the time. And not alone in our large cities. Only that in the rural districts they are scattered and proportionably less mischievous. A filthy village hut is bad, but a dirty street is worse. And like begets like everywhere. In our large cities, the filthy huts and dirty streets partake of the nature of their congener—the cholera—multiply infinitely. Want of pure air, want of pure water, want of light, want of drains, want of sewers, and in cities want of salubrious exercise grounds, combine to produce dampness, stagnation, uncleanness, misery, disease, and death.

The more numerous such places, and the more crowded they are, the worse they are, and the more subject to cholera. And here, too, lie the seeds of crime, the materials of mobs and riots, the enemies of order, the instruments of the demagogue and the tools—ay, sometimes the school—of politicians.

The instinctive impulse to flee from such sickening influences suggests the remedy. For the present, doubtless something can be done out of the usual order to abate certain "localizing causes"; but the root of the evil is not to be reached by the untenanting of the poor, unfortunate cellar denizens. It is against *such facilities*, that not only he who avails himself of them, but every community should be protected. The owners and abettors of degrading habitations, the nurseries of crime, disease, and they should be treated accordingly—as common enemies of mankind.—*Sanitarian.*

